# Spectroscopic Identification of Bacteria Resistance to Antibiotics by Means of Absorption of Specific Biochemical Groups and Special Machine Learning Algorithm

**DOI:** 10.3390/antibiotics12101502

**Published:** 2023-09-30

**Authors:** Claudia P. Barrera-Patiño, Jennifer M. Soares, Kate C. Branco, Natalia M. Inada, Vanderlei Salvador Bagnato

**Affiliations:** 1 São Carlos Institute of Physics, University of São Paulo, Avenida Trabalhador São-Carlense n° 400, Parque Arnold Schimidt, São Carlos 13566-590, SP, Brazil; jennifer.soares@usp.br (J.M.S.); kateblanco@ifsc.usp.br (K.C.B.); nataliainada@ifsc.usp.br (N.M.I.); 2 Biomedical Engineering, Texas A&M University, 400 Bizzell St, College Station, TX 77843, USA

**Keywords:** *Staphylococcus aureus*, FTIR spectroscopy, antibiotic-resistant bacteria, amoxicillin induced, gentamicin induced, erythromycin induced, machine learning algorithms

## Abstract

FTIR (Fourier transform infrared spectroscopy) is one analytical technique of the absorption of infrared radiation. FTIR can also be used as a tool to characterize profiles of biomolecules in bacterial cells, which can be useful in differentiating different bacteria. Considering that different bacterial species have different molecular compositions, it will then result in unique FTIR spectra for each species and even bacterial strains. Having this important tool, here, we have developed a methodology aimed at refining the analysis and classification of the FTIR absorption spectra obtained from samples of *Staphylococcus aureus*, with the implementation of machine learning algorithms. In the first stage, the system conforming to four specified species groups, Control, Amoxicillin induced (AMO), Gentamicin induced (GEN), and Erythromycin induced (ERY), was analyzed. Then, in the second stage, five hidden samples were identified and correctly classified as with/without resistance to induced antibiotics. The total analyses were performed in three windows, Carbohydrates, Fatty Acids, and Proteins, of five hundred spectra. The protocol for acquiring the spectral data from the antibiotic-resistant bacteria via FTIR spectroscopy developed by Soares et al. was implemented here due to demonstrating high accuracy and sensitivity. The present study focuses on the prediction of antibiotic-induced samples through the implementation of the hierarchical cluster analysis (HCA), principal component analysis (PCA) algorithm, and calculation of confusion matrices (CMs) applied to the FTIR absorption spectra data. The data analysis process developed here has the main objective of obtaining knowledge about the intrinsic behavior of *S. aureus* samples within the analysis regions of the FTIR absorption spectra. The results yielded values with 0.7 to 1 accuracy and high values of sensitivity and specificity for the species identification in the CM calculations. Such results provide important information on antibiotic resistance in samples of *S. aureus* bacteria for potential application in the detection of antibiotic resistance in clinical use.

## 1. Introduction

Since the discovery of penicillin’s antimicrobial ability by Fleming in 1928, antibiotics have significantly extended the human lifespan and are regarded as one of the most crucial medical breakthroughs of the 20th century. Therefore, they are unquestioningly used for the treatment of numerous life-threatening infectious diseases [1,2,3,4]. The current excessive and inappropriate use of antibiotics in both humans and livestock has resulted in a surge of antibiotic-resistant bacteria, and over the past few decades, the emergence of multidrug-resistant organisms (MDROs) has escalated into a worldwide crisis [4,5]. This fact regained global attention amidst the worldwide research aimed at managing and combating COVID-19 disease. During that period, it became evident that bacterial identification techniques include some challenging processes [6], thus implying that a rapid and quantitative detection of bacterial antibiotic resistance is of high significance for the prevention and treatment of infections and understanding of drug-resistant mechanisms [7].

Some species known as ESKAPE (*Enterococcus faecium*, *Staphylococcus aureus*, *Klebsiella pneumoniae*, *Acinetobacter baumannii*, *Pseudomonas aeruginosa*, and *Enterobacter* spp.) are responsible for a large number of hospital-acquired infections with high levels of resistance to several antibiotics commonly used in the treatment of bacterial infections [8]. In this context, machine learning algorithms have been increasingly used as an effective tool to identify patterns and predict bacterial resistance. The identification of resistant strains aids in the optimization of antibiotic administration, offering precise insights into bacterial susceptibility to several drugs available [9].

In recent years, different and new techniques have been adopted for the identification of signals from systems that develop susceptibility to antibiotic resistance [4,5,6,10,11,12,13,14,15,16]. Understanding the mechanisms and genotypic/phenotypic alterations behind resistance is critical for the further development of new drugs and often requires incorporation of innovative research strategies [5,11]. Also, it is a priority to shorten the time between bacterial identification and a diagnosis of antibiotic resistance [4,5]. Research on the topic is relevant in the health, industrial, scientific, and military systems worldwide [5,11,12,13,14]. An example is the need for a rapid and simple method for the accurate identification of Methicillin-resistant *S. aureus* (MRSA) [12,16].

Currently, the antibiotic susceptibility test (AST) is based on the following three methods: disk diffusion, gradient diffusion, and agar/broth dilution [4]. The common techniques for the identification of bacteria are the culture-based one, polymerase chain reaction (PCR), enzyme-linked immunosorbent assay (ELISA), mass spectroscopy [10,14], and DNA sequencing [16]. Novel techniques such as flow cytometry [4], matrix-assisted laser desorption ionization-time of flight mass spectrometry (MALDI-TOF MS), weighing bacteria with and without antibiotic treatment by vibration, isothermal microcalorimetry, rotating magnetic ligand-modified beads, and micro- and nano-droplets [4] have been devised to advance the pursuit of rapid antibiotic susceptibility testing (AST). Ciloglu et al. [10] demonstrated that positively charged AgNPs provide a strongly enhanced Raman signal due to electrostatic aggregation. However, all such techniques are currently under development and have not replaced the traditional AST methods [4]. Obtaining a response to the detection challenger requires the adoption of new techniques that combine laboratory and computational tools, implying a further advancement in optical and spectroscopy detection tools and machine learning algorithms toward more user-friendly, safe, rapid, and cost-effective detection [13,15]. 

Spectroscopic techniques show promise as valuable tools in biomedical diagnostics [4,5,7,11,13,14,15,16], and combinations of atomic force microscopy-infrared spectroscopy (AFM-IR) and chemometric analysis, as well as Fourier transform infrared spectroscopy (FTIR) and new statistical classification methods have been developed [11]. Infrared absorption microscopy provides detailed information on the spatial distribution of the chemical composition at the molecular level [5,11]. The FTIR spectrum of a given molecule is highly specific due to the dense chemical information contained in a single spectrum [12]. Spectral information requires advanced data processing algorithms to capture minor differences in the data acquired in studies focused on identifying resistance susceptibility fast and safely [6]. These aim to identify changes in the chemical composition of bacteria associated with the development of resistance [5,11,12]. FTIR along with identification techniques such as microbial culture and genetic sequencing have been used as an auxiliary tool for microbial diagnosis. However, they have caused the emergence of a recent challenge related to the interpretation of similar spectra [10]. Spectroscopic-based pattern recognition implies the adoption of machine learning algorithms (MLAs). Previous research has shown that MLAs have a high accuracy in distinguishing very similar spectra from different strains [10,16]. This identification is essential to successfully monitor and initiate the necessary treatment and medication [6]. 

In this context, supervised and unsupervised machine learning algorithms were incorporated in this study for the data analyses of the FTIR absorption spectra obtained from samples with and without induced antibiotic resistance. The aim was to contribute with a safe tool for the recognition of important information to be used in health science. The focus was on this research field for developing the methodology from previous and recent research work with excellent results [1,2,3]. A strategy for the analyses and processing of data from the FTIR spectra of *S. aureus* bacteria was also conceived here to develop this study. Each step of this study was accurately conducted and designed for the acquisition and processing of information by using the aforementioned spectroscopic tools [4,5,6,7,10,11,12,13,14,15,16]. This motivated us to study how to refine the analysis and classification of the FTIR absorption spectra obtained from *S. aureus* samples, with the implementation of MLAs. In the first stage, the system conformed by the specified species groups, namely, Control, Amoxicillin induced (AMO), Gentamicin induced (GEN), and Erythromycin induced (ERY), was analyzed with the HCA and PCA unsupervised machine learning algorithms [17,18,19,20,21,22,23,24,25,26]. In the second stage, five hidden samples were identified and correctly classified as with/without resistance to induced antibiotics with CM [16] supervised machine learning algorithms. The total analyses were carried out in three windows, Carbohydrates (900–1200 cm^−1^), Fatty Acids (2800–3100 cm^−1^), and Proteins (1500–1800 cm^−1^), in a sample of five hundred spectra acquired following the procedure report by Soares et al. in [1,3] with the resistance-induced strains protocol by Soares et al. in [2] and processed in their entirety here in MATLAB [27] and with machine learning algorithms in R free software (version 4.2.3, 15 March 2023) [28,29,30,31,32,33,34,35,36] by means of our own codes developed for this study. 

## 2. Results

### 2.1. Acquisition, Cleaning, and Processing of Data from FTIR Absorption Spectra

*S. aureus* is a Gram-positive bacterium with a thick peptidoglycan layer at the outermost cell wall. Peptidoglycan biosynthesis is an excellent target for most antibiotics [6]. This study explores the identification of antibiotic resistance in *S. aureus* bacteria through a data analysis of the FTIR absorption spectra derived from samples belonging to the Control, AMO, GEN, and ERY groups [2] and five hidden samples for the identification of antibiotic resistance susceptibility. Five hundred FTIR absorption spectra were acquired following the protocol developed to acquire the samples of *S. aureus* in [1,2,3]. 

**Figure 1:**
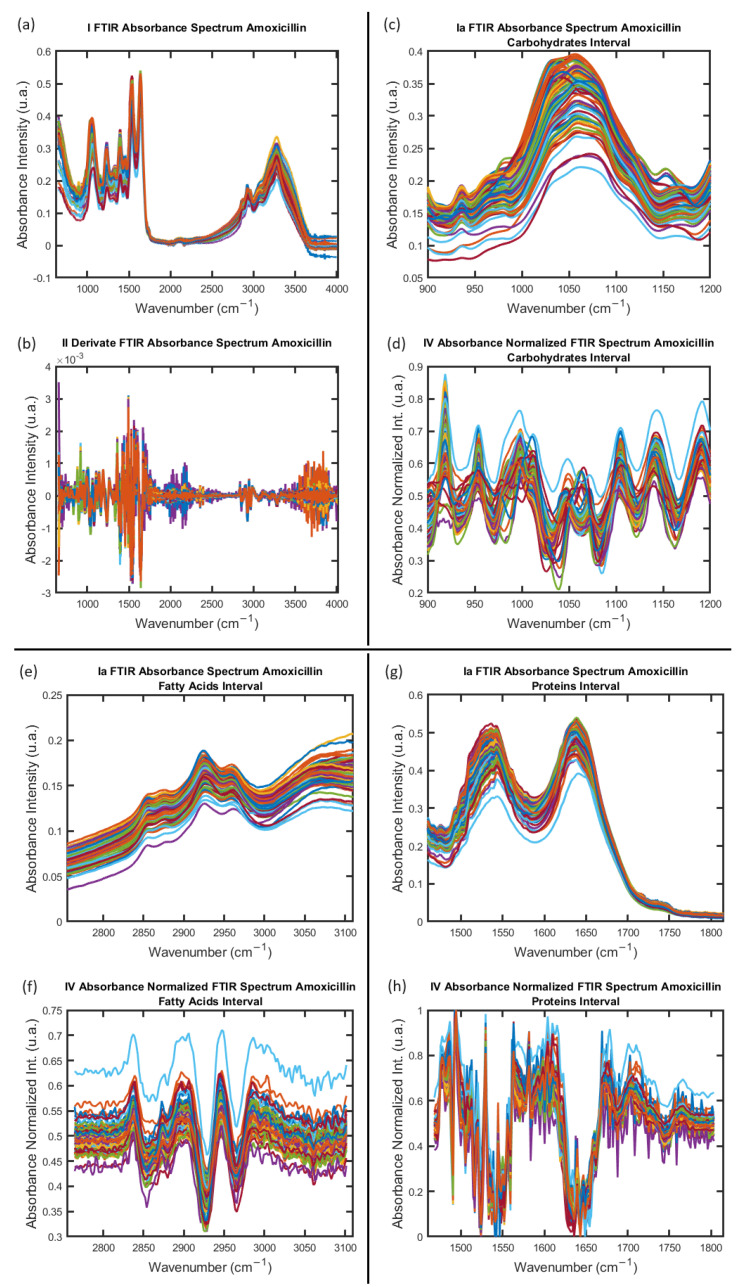
FTIR absorbance spectrum obtained for *S. aureus* samples with Amoxicillin−induced Amoxicillin−induced antibiotic resistance. (**a**,**b**) One hundred FTIR absorbance spectra and second derivative calculation. (**c**,**d**) Carbohydrates interval window in record FTIR absorbance spectra and processed spectra with absorbance normalized intensity for this interval group. The same process is shown for Fatty Acids interval window (**e**,**f**) and Proteins interval window (**g**,**h**). Spectra shown in (**a**) were acquired following the procedure report by Soares et al. in [1,3] with resistance-induced strains protocol by Soares et al. in [2]. Spectra (**b**,**d**,**f**,**h**) were obtained to apply the steps of the data process in [3] implemented here in MATLAB [27] by means of own codes developed for this study.

Once the FTIR absorption spectra of the samples were obtained, the cleaning process of the entire sample with 500 spectra was started. The first step of the data analysis process was developed in MATLAB [27] following the protocol introduced in [37] (more details in Section 4). Figure 1 shows the result of this first process. Subsequently, the representative spectra sample for each species was identified from the outcomes of the dendrogram algorithm, establishing the foundation for the implementation of the MLA for a statistical analysis. The data analysis of the FTIR absorption spectra started with the hierarchical cluster analysis (HCA) and then the principal component analysis (PCA) and finished with the confusion matrix (CM) calculations; all these steps were developed in R software [27,28,29,30,31,32,33,34,35,36]. All the details about the calculations step can be found in methods in Section 4. The data analysis process developed here has the main objective of obtaining knowledge about the intrinsic behavior of the *S. aureus* samples within the analysis regions of the FTIR absorption spectra. 

### 2.2. HCA and PCA Applied for the Study of FTIR Absorption Spectra 

The results were obtained with the implementation of the machine learning unsupervised algorithms in the spectrum data analyses in each step, and then in the data analyses of the FTIR absorption spectra, different statistical methods were implemented, depending on the data characteristics to be analyzed in each step in the total analyses process. We developed the spectra data analyses with the identification of the similarities and dissimilarities in the FTIR absorption spectra samples with machine learning unsupervised algorithm tools like the dendrogram, PCA-center, and PCA in the free software R [28,29,30,31,32,33,34,35,36]. 

The first step to obtain the individual spectra contribution was implemented in the study statistical tools provided by means of the dendrogram with the implementation of the Euclidean distance and single-linkage method in R (Figures S1–S3 in Supplementary Materials). The PCA-center (results in Figures S4–S6 in Supplementary Materials) and PCA use the calculation of the principal component percentage variance by means of the implementation of the default functions in R. For the PCA, the statistical analyses are shown directly on the figure. 

The dendrogram analyses (results in Figures S1 and S3 of the Supplementary Materials) were implemented to identify the spectral similarities in the species groups and acquire a comprehensive understanding of each individual spectrum’s contribution to the PCA. Both unsupervised machine learning algorithms [17,18,19,20,21,22,23,24,25,26] were implemented in R [28,29,30,31,32,33,34,35,36]. The spectra were classified by HCA. A statistical tool in the dendrogram calculation provided a spatial organization in the entire spectra sample by agglomerative clustering [22,23,24,25,26,38], enabling the visualization and identification of the FTIR absorption spectra groups with statistical significance [20,24,25,26,38,39,40,41,42]. The approach provides a valuable means to structure the input group of spectra for a PCA within the scope of the antibiotic resistance study.

PCA was used to reduce the primary FTIR spectrum sample data into two spatial dimensions representations. The correlations between the original data and each principal component must be computed for the interpretation of each component [43,44,45,46,47,48,49]. The PCA-center was then conducted for the three windows, namely, the Carbohydrates, Fatty Acids, and Proteins, for each species group studied, the Control, AMO, GEN, and ERY. First, the PCA-center calculations, which refer to obtaining the spatial representation of the mass center for one hundred spectra in each species group, were performed for the visualization and observation of the intrinsic statistical attributes from the scale results of the spectra sample. Figures S4–S6 in the Supplementary Materials show the visualization of the PCA-center’s results and the spatial distribution of the four species in each window interval group. It also displays the statistical significance of the components, which helps with data interpretation.

**Figure 2:**
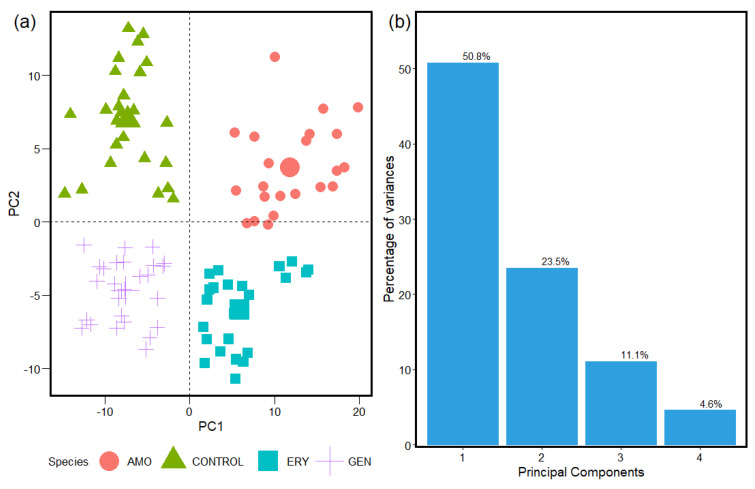
(**a**) PCA spatial distribution Control, Amoxicillin−induced, Gentamicin−induced, and Erythromycin−induced species. (**b**) Results of statistical significance calculations for the Carbohydrates interval in FTIR spectrum of *S. aureus*.

**Figure 3:**
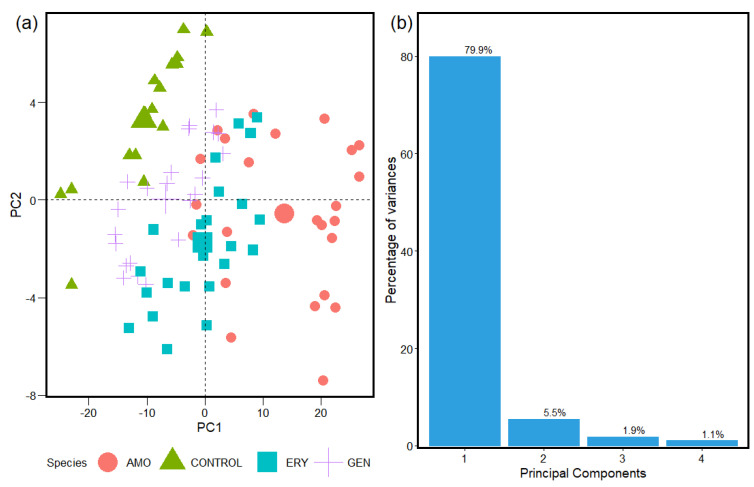
(**a**) PCA spatial distribution of Control, Amoxicillin−induced, Gentamicin−induced, and Erythromycin−induced species. (**b**) Results of statistical significance calculations for the Fatty Acids interval in FTIR spectrum of *S. aureus*.

**Figure 4:**
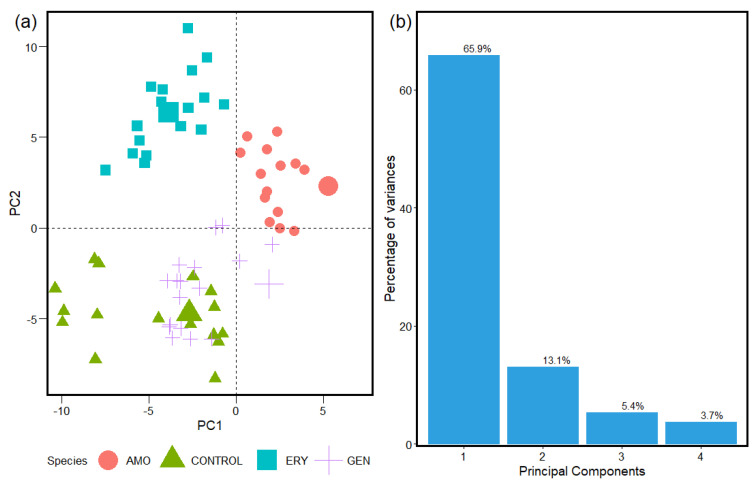
(**a**) PCA spatial distribution of Control, Amoxicillin−induced, Gentamicin−induced, and Erythromycin−induced species. (**b**) Results of statistical significance calculations for the Proteins interval in FTIR spectrum of *S. aureus*.

Figure 2, Figure 3 and Figure 4 show the statistical results for the FTIR absorption spectra of *S. aureus* from the analyses of the unsupervised machine learning PCA algorithms. The spatial distribution obtained with the PCA calculation helps the interpretation and identification of the cluster formed by the similarities in the samples from the with and without antibacterial resistance groups.

### 2.3. Confusion Matrix Results Applied in the Study of FTIR Absorption Spectrum

The use of MLA for data analyses in biomedical applications has increased in recent years. In particular, the use of CMs [16] has been positioned as a high-performance tool with a high level of security in the results obtained. For this reason, here, the CM was implemented in the methodology to develop the identification and classification of five hidden samples of *S. aureus* with/without resistance to the antibiotic-induced samples, from the analyses of one hundred FTIR spectra. All the calculations were developed without prior knowledge of the antibiotic susceptibility or non-resistance developed in the samples.

The CM was implemented in the study of the antibiotic resistance in *S. aureus* bacteria from the analyses of the FTIR absorption spectra for four species groups, the Control, AMO, GEN, and ERY, in the three windows intervals, the Carbohydrates, Fatty Acids, and Proteins, in the FTIR absorption spectra samples. The code was made with the default functions in R [16,28,29,30,31,32,33,34,35,36], and the input data were taken from the spectrum data obtained from the results of the data analyses performed with the dendrograms and PCA machine learning algorithms.

In the first step, the spectra input file for the four species, the Control, AMO, ERY, and GEN, introduced the data into the CM construction. Then, the data analyses began with the process of classification in the training group and testing group. The last step was automatically conducted by the CM; in all the processes, we did not develop any manual classification of the data. When the CM accuracy rose to value 1, after trying different testing and training percentage groups from the input data, we obtained a safe way to identify the species in the confusion matrix calculation results. At this point, we were sure that the data and configuration of our CM were suitable to start the data analyses to identify and classify the five hidden samples with/without resistance to the antibiotic-induced samples from the FTIR absorption spectra. Then, we processed the data in the next step, and we introduced the FTIR absorption spectra data from the hidden samples into the matrix confusion calculation.

**Figure 5:**
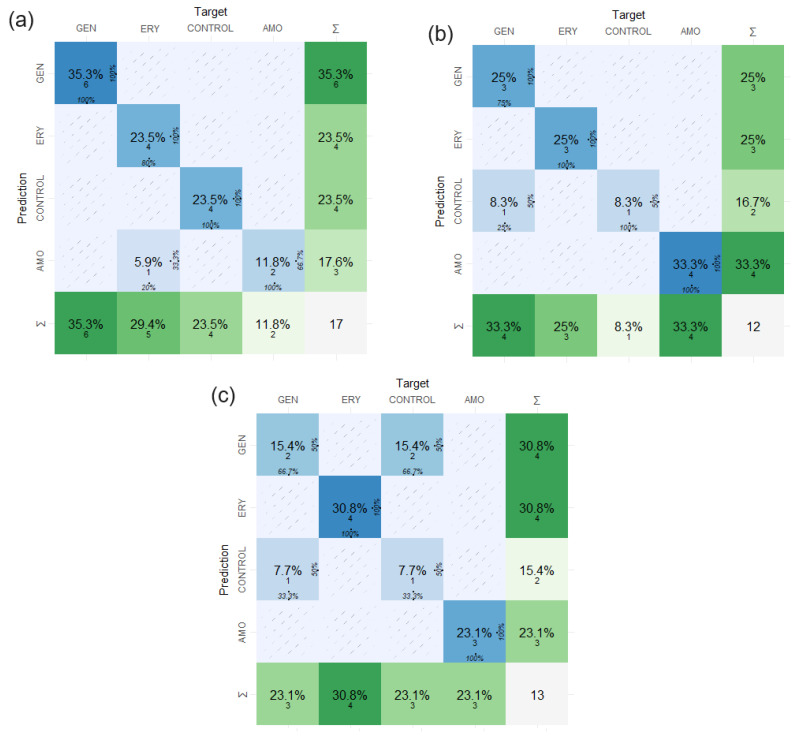
Confusion matrix results obtained with dataset from FTIR absorption spectra from dendrograms and PCA. From the input data, 80% of data were chosen to train the models and 20% to test in all the matrix confusion calculations. To follow the results obtained, (**a**) Carbohydrates results in 0.941 accuracy, (**b**) Fatty Acids results in 0.917 accuracy, and (**c**) Proteins results in 0.772 accuracy for interval groups in the FTIR absorption spectra. In the table are the statistics of the calculations; the prediction results are between 0.8 and 1 of accuracy.

The MC helped us process the predicted outcomes of the antibiotic resistance in the hidden samples due to the excellent performance of the model performed with the process implemented to make the confusion matrix in this study. We obtained the results from the MC of the first system, the Control, AMO, ERY, and GEN, in the interval groups: Carbohydrates, results in 0.941 accuracy; Fatty Acids, results in 0.917 accuracy; and Proteins, results in 0.772 accuracy. Figure 5 introduces the value results from the MC to the evaluated windows intervals in the FTIR absorption spectra samples of *S. aureus*. 

The statistics elements in the confusion matrices of the accuracy, sensitivity, and specificity follow the equation expressions (1)–(3), respectively,

(1) $$accuracy=\frac{\left(TP+TN\right)}{\left(TP+TN+FP+FN\right)},$$

(2) $$sensitivity=\frac{TP}{\left(TP+FP\right)},$$

(3) $$specificity=\frac{TN}{\left(TN+FP\right)},$$

with FN: False Negative, FP: False Positive, TN: True Negative, and TP: True Positive.

These expressions were applied in all the CM calculation results reported in this study. The results for the calculations of the five hidden samples from the CM results for antibiotic identification are shown in Table 1.

The analyses of the MC results and statistical parameters in the calculation are introduced in Figure S7 (results in Supplementary Materials) to exemplify and show the way in which the MC reported the results calculation. The CM results of the identified and classified resistance to the antibiotic-induced samples from the FTIR absorption spectra in the hidden samples SK in the Carbohydrates interval window for the species Control, AMO, ERY, and GEN are shown in Figure S7 (Supplementary Materials). In this calculation, 80% of the data were selected to train the models and joined 20% of the data for testing in the MC. Figure S7 shows the CM plot results, the prediction results table from the confusion matrix with 0.938 accuracy, and the CM calculation table with the statistical parameter results for the accuracy, sensitivity, and sensibility obtained for the hidden sample SK. With the MC statistical parameter calculations reported for each hidden sample calculation (Figure S7c), Table 1 was constructed for the five hidden samples, the SK, SW, SX, SY, and SZ, in the groups Control, AMO, ERY, and GEN, for the interval groups, Carbohydrates, Fatty Acids, and Proteins, in FTIR absorption.

Here, we report in Table 2 the accomplishment in implementing the CM calculations to identify the antibiotic resistance from the FTIR absorption spectra for the five hidden samples. The entire process developed in this study for the hidden samples was performed without previous knowledge or information about the species involved in the MC calculation from the FTIR absorption spectra in the windows groups, Carbohydrates, Fatty Acids, and Proteins. From the CM-obtained results, it was possible to detect, in a safe way (Table 1 and Table 2), three samples with antibiotic resistance to AMO, ERY, and GEN; one without antibiotic resistance; and one with MRSA.

The confusion matrix identification results reported in Table 1 and Table 2 let us define in a safe way the identification of antibiotic resistance from the FTIR absorption spectra in the samples of *S. aureus*. The accuracy calculation value reported from the CM results is between 0.70 and 1, and the sensitivity and specificity values are between 0.7–0.95 and 0.861–0.987, respectively (Table 1). According to the results, a CM is an accurate MLA tool for the identification of samples with/without antibiotic resistance in *S. aureus*. All the CM calculations were developed and implemented to safely and carefully obtain intrinsic information from the hidden samples.

## 3. Discussion

The increasing number of antimicrobial-resistant bacteria is a major threat to global health, and the identification of resistant strains is a critical topic in different fields of research [50]. Antimicrobial resistance occurs when bacteria develop the ability to resist the effects of antibiotics, thus causing more severe infections, prolonging hospitalization, and increasing both treatment costs and the risk of mortality [51]. Antimicrobial resistance mechanisms can occur through the mutations or transfer of genetic material between different bacterial species and through the artificial selection of strains with an excessive and inappropriate use of antibiotics [52]. 

MLA has been employed in the generation of newly discovered and new research topics in different knowledge areas, like life sciences, the biological system, biosciences, bioinformatics, biomedical and biomechanics; it is due to its versatility and accuracy [10,12,29,42,53,54]. In the current study, MLA methodology was implemented for the identification and classification of antibiotic resistance in *S. aureus* samples with the analyses of the FTIR absorption spectra. 

The biomolecules detected in the FTIR are mainly those present on the surface of the bacterial cell [55]. Figure 1 displays four hundred FTIR absorbance spectra for the sample, evidencing the challenge for the identification and determination of the spectra similarities and dissimilarities toward obtaining the statistical significance of individual spectra [29,56]. The MLA results from the investigation of the antibiotic resistance in the FTIR absorbance spectra of *S. aureus* in the Carbohydrate, Fatty Acids, and Proteins windows for the Control, Amoxicillin-induced (AMO), Gentamicin-induced (GEN), and Erythromycin-induced (ERY) species proved to be useful for the data analysis of the spectroscopy database shown in Figure 1. Moreover, the HCA and PCA applied to the FTIR absorption spectra dataset enables the identification of the individual contributions and intrinsic behavior in the samples of each data (spectra) and in each window of the four species. 

Such connections help the classification of samples, because spectra dissimilarities lead to an increase in the separation distance and a change in the cluster composition, i.e., the distance between the objects in the Euclidean space increased with the spectra dissimilarities [18,19,20,21,22,23,24,25,26,57], and clustering by similarity distance metric and linkage criterion [29,38]. 

From the PCA-center (results in Figure S4–S6 of the Supplementary Materials), the results of the analysis in the first principal component indicate a significance of 62.4% for the Carbohydrate window, 95.5% for the Fatty Acids window, and 59.1% for the Proteins window. The PCA-center statistical significance gets to 81.7%, 98.8%, and 83.8% between the first and second component for the same interval windows groups. Then, the statistical significance is directly related to and dependent on the variance value of the sample. The PCA-center spatial representation promotes a clear visualization of the calculation results and the general behavior of the FTIR absorbance spectra in the four species samples groups for the studies of the susceptibility to the antibiotics. 

The statistical reports, shown in Figure 2, Figure 3 and Figure 4, from the classification spectra process obtained with the HCA methods used before to implement the PCA determine a representative sample to be analyzed with accuracy. This is reflected in the statistical information obtained from the PCA for the species groups studied for antibiotic resistance in *S. aureus* with the spectra sample selected and associated to the statistical weight of the entire sample of the FTIR absorption spectra. This is due to between ~70% and 80% of the sample analyses being contained in the two first components for the three windows. That brings a safe report from the methods implemented to the classification of spectra and data analyses tools implemented in the machine learning algorithms used in this study. 

The PCA calculation (Figure 2, Figure 3 and Figure 4) results from the spectra samples classification were obtained with the HCA methods (results in Figures S1–S3 of the Supplementary Materials). The spatial distribution of the Control, Amoxicillin-induced, Gentamicin-induced, and Erythromycin-induced species and the results of the statistical significance calculations for the Carbohydrates, Fatty Acids, and Proteins intervals in FTIR’s spectrum of *S. aureus* with values of 74.3%, 85.4%, and 79% between the first and second principal components of the analyzed samples are displayed. The different responses obtained for *S. aureus* in the three windows implies the bacteria interact with the antibiotic species in different ways. Consequently, some convergent strategic solutions are introduced to break the reproduction of resistant bacteria. FTIR absorption spectra can vary due to the biochemical composition of a bacterium, which can be modified in function of the culture conditions under which it grows. Therefore, FTIR can be used not only to classify bacteria at broad levels such as genus but also to identify the degrees of antimicrobial resistance.

The data analyses started in the Carbohydrates windows, obtaining a very clear response on the organization of data for linearities, the cluster formation, and the spatial distribution in the 2D space for each species group studied (Figure 2). All the statistical contributions and information from the samples in that window are contained in the four principal components. The results from the PCA for the studies of the antibiotic response in the Carbohydrates group window are promising for the implementation in studies with FTIR absorption spectra. This is in association with the fact that Carbohydrates are present in glycoproteins and in the very constitution of the cell wall, which is mainly composed of peptidoglycan, a polysaccharide of chains of Carbohydrates and amino acids [58]. Carbohydrates are inherent to metabolic pathways and, despite not being direct targets of antibiotics, they are affected by beta-lactam antibiotics, which inhibit the synthesis of peptidoglycan and can lead to a loss of cell wall integrity [59]. Resistance associated with beta-lactams is related to changes in the constitution of the cell wall [60]. As shown in Figure 2a, the data referring to AMO-induced *S. aureus* bacteria are located in different quadrants in relation to the Control group, and ERY-induced *S. aureus*, which, as a macrolide, affects the synthesis of Proteins in bacteria by blocking the binding of amino acids and tRNA [61]. It can also indirectly affect the synthesis of Carbohydrates, because the enzymes involved are generally encoded by genes that are in the same operon as those that encode Proteins. 

Figure 3 shows the results of the Fatty Acids, which are constituents of the bacterial plasma membrane. In the Fatty Acids window, the results from the PCA report excellent statistical significance associated to the study of the FTIR spectrum of the *S. aureus* spectra in response to antibiotics resistance. Figure 3a shows data that AMO-induced bacteria are the most separated ones from the other groups analyzed. Because amoxicillin interferes with the synthesis of the bacterial cell wall, it can affect the bacterial metabolism indirectly, including the synthesis and use of Fatty Acids. ERY-induced and GEN-induced bacteria highly overlap, because both act on the ribosome despite being in different regions [62]. They permeate all quadrants, overlapping with the regions of the Control and AMO-induced bacteria. Therefore, ERY- and GEN-induced resistance can lead to uncertain conclusions on the classification between sensitive and resistant in the analysis of the overall data. This spatial distribution for the Fatty Acids windows is inherent to the spectra contained in them; this implies some special deterministic behavior in this region for the samples in which antibiotic resistance was induced in *S. aureus* bacteria. According to the statistical calculation obtained by the PCA for the Fatty Acids window (Figure 3b), 80% of the samples were classified in the first component, which may be associated with the structural conformation of the samples in that window. Although Fatty Acids are not the direct target of Amoxicillin, Erythromycin, and Gentamicin, such antibiotics can affect their metabolic pathways [59]. 

The results from the PCA in the Proteins interval window for the four species (Figure 4) in the FTIR absorbance spectra of the *S. aureus* display the spatial distribution, which is a result of the intrinsic properties of the sample in the window studied, and also supply statistical contribution information of the spectra analyzed. The results can be associated with the fact biomolecules such as Proteins and enzymes are affected by different types of antibiotics [63] and, consequently, the spectra in this window display a different behavior in relation to the one exhibited in both previous group windows. Macrolides and aminoglycosides have an inhibitory action on Protein synthesis, directly targeting the Protein synthesis machinery of bacteria, and the ribosome, and blocking the translation of essential Proteins and enzymes for cell survival [62,64]. Amoxicillin blocks the transpeptidation reaction by binding to Penicillin-Binding Proteins (PBPs) [65]. Such different relationships of antimicrobials with Proteins result in different distributions among the quadrants for the analyzed groups in the PCA, with only some data from Gentamicin present in the quadrant of the Control group. 

This study revealed a profile for the identification of MRSA, a pathogenic bacterium that has developed resistance to the methicillin antibiotic and beta-lactams. It shows modifications in its structural components as a consequence of the effects of different antimicrobial resistance factors. A change in the PCA was observed for the MRSA profile of lipids, Proteins, and Carbohydrates compared to the non-antibiotic resistant strains. Changes in both composition and structure of lipids occur especially in cell membranes and in membrane fluidity, involving an increase and a reduction in saturated and unsaturated Fatty Acids, respectively, resulting in the thickening of the membrane which affects the permeability of the membrane, making it difficult for the entry of antibiotics in bacterial cells. On the other hand, the modification of the peptidoglycans (Carbohydrates) observed in the PCAs is directly related to methicillin resistance and the modification of the peptidoglycan precursors that influence the cell wall. Another point that is part of the resistance profile studied is the presence of altered Proteins, which interfere with the binding of beta-lactam antibiotics. An explanation for such changes is the genetic modifications resulting from gene expression, which is part of the peptidoglycan synthesis system in the bacterial cell wall, and the production of enzymes, such as hemolysins, leukocidins, and exotoxins.

The results of the identification and classification of the samples with/without resistance-induced antibiotics (results in Figure 5 and Table 1 and Table 2) have confirmed that the confusion matrix (CM) enables a safe calculation and a highly accurate identification of *S. aureus* samples. The CM calculation from one hundred FTIR absorption spectra samples to five hidden samples report accuracy values between 0.70 and 1, with sensitivity and specificity values between 0.80–0.93 and 0.92–0.98, respectively (Table 2). The confusion matrix prediction results led to the successful identification of antibiotic resistance from the FTIR spectra samples of *S. aureus*. They are associated with the intrinsic classification of the biologic, chemical, and physical properties of the system, which can be obtained by the MLA applied to the spectra windows of Carbohydrates, Fatty Acids, and Proteins for the three antibiotic species and Control groups, thus suggesting an inherent response of *S. aureus* to the antibiotics in each FTIR spectra window studied. All the CM calculations were developed and implemented to obtain the intrinsic information from the hidden samples in a safe and careful way and to check with rigor. 

The results from the implementation of the supervised and unsupervised algorithms for the identification of antibiotic resistance from the FTIR absorbance spectra enabled accurate data organization by statistical weight and a clear visualization of the organization for each species. According to the statistical reports, the methods and principles applied here in the data analyses were successfully conducted and, therefore, can be extended to other systems and to another bacteria of interest.

## 4. Materials and Methods

### 4.1. FTIR Absorption Spectrum of S. aureus Acquisition and Data Process in MATLAB

FTIR absorption spectra of *S. aureus* were acquired following the procedure reported by Soares et al. in [1,3] with resistance-induced strains protocol by Soares et al. in [2], and data processing code was developed following the steps of the protocol of Naumann [37] in MATLAB (R2021b) [27]. 

Nine hundred FTIR absorption spectra of *S. aureus* were acquired with FTIR equipment by Attenuated Total Reflection (ATR) on the Agilent Cary 630 FTIR Spectrometer^®^ instrument in the wavelength range of (650–4000) cm^−1^, acquisition time of 20 min to each spectrum record. In total, 800 spectra for the species group and 100 spectra for hidden samples were recorded. Spectra species group was acquired two times in order to guarantee measurement; due to no representative variances in the spectra sample being obtained, the first records in all samples analyzed were chosen.

Five hundred FTIR absorption spectra of *S. aureus* were analyzed in the study in the following way: 400 spectra for species group, Control (100 spectra), Amoxicillin-induced (AMO) (100 spectra), Gentamicin induced (GEN) (100 spectra), and Erythromycin induced (ERY) (100 spectra); and 100 for hidden samples (100 spectra) with 20 for each one. 

Data analyses of the FTIR absorption spectrum of *S. aureus* bacteria from each species mentioned before received the same data process; these were processed according to the next steps:

(i) FTIR absorption spectra acquisition one by one [1].
(ii) Calculation of the second derivative for each spectrum individually for each species group of one hundred FTIR absorption spectra [37]. It was performed by means of the implementation of the second-order difference of dataset in MATLAB [27]. That means that each point in the spectrum dataset was associated to one vector (λ_1_, I_1_). It corresponded to one array formed by the wavelength value and its correspondent FTIR absorption intensity record value. Then, each vector was processed to compute the second-order difference. This method also allows for calculating differences between adjacent elements. The entire calculation process was developed with default functions available in MATLAB [27].
(iii) Normalization by maximum value of FTIR absorption intensity [37]; process conducted in each spectrum individually.
(iv) Extract the window interval group; it conformed the array of one hundred FTIR absorption spectra intensity with the same wavelength values [37].

To illustrate the spectra obtained in each step, Figure 1 displays the spectra obtained from the calculations process for the species group Amoxicillin induced (AMO) for the three windows intervals: Carbohydrates, Fatty Acids, and Proteins. The array obtained from the data spectrum analyses performed in MATLAB [27] was the input data worked into the data analyses with machine learning algorithms in R Project for Statistical Computing (version 4.2.3, 15 March 2023) https://www.r-project.org/ (accessed on 15 March 2023) [28,29,30,31,32,33,34,35,36]. 

### 4.2. Supervised/Unsupervised Machine Learning Algorithms Applied to Spectrum Analysis

Implementations of spectral tools for bacterial analyses offer advantages, such as rapid test, easy handling, nondestructive data acquisition, and fingerprint detection [16]. Spectra from surface-enhanced Raman spectroscopy (SERS) can be reduced to a few independent latent variables that account for most variability in the original dataset by multivariate statistical analyses [15]. Current supervised classification machine learning algorithms were implemented toward that goal. Principal component analysis (PCA), hierarchical cluster analysis (HCA), linear discriminant analysis (LDA), k-nearest neighbors (kNN), and linear support vector machines (SVM) have provided optimal results, according to research reports [5,7,10,16]. Deep learning [16], convolutional neural network (CNN) [16], and deep neural network (DNN) have been adopted for discriminating antibiotic-resistant bacteria with the use of surface-enhanced SERS [6,13,14]. Supervised classification techniques can be used especially for the strain level classification [10], whereas unsupervised machine learning technique is adopted for anomaly detection and works well in multivariable datasets [10]. The confusion matrix of kNN classifier has been shown to achieve the highest accuracy among other methods [10]. 

### 4.3. Machine Learning Algorithms

This subsection describes the steps followed in the data processing and analysis, which include sample preparation, acquisition of FTIR spectra record for the four species and hidden samples, data loading, classification, preprocessing for the three window groups in the four species, clustering for spectra selection, dimensionality reduction, data visualization, and sample evaluation and validation of antibiotic resistance by confusion matrix. Available functions were used in MATLAB (R2021b) [27] and R Project for Statistical Computing (version 4.2.3, 15 March 2023) [28,29,30,31,32,33,34,35,36]. All codes were implemented for the development of the spectra data analysis techniques for the antibiotic resistance study of *S. aureus* samples. 

For this work, no novel software was developed. In this work, the functions contained and supplied by default in the software versions installed were used. We employed these established functions to create our own codes sources; we implemented them to develop the spectra data analysis techniques for antibiotic resistance study of *S. aureus* samples. 

In the following, we introduce the general information about owner code sources construction (more details in the Supplementary Materials in the section Code and programming). The first step was the introduction and code implementation of the protocol of [37] for the organization of 500 spectra data from FTIR vibrational spectroscopy for the four species, Control, AMO-induced, ERY-induced, and GEN-induced bacteria, and 100 spectra for hidden samples, in MATLAB (R2021b) [27]. The steps included (i) tests for spectral quality, (ii) application of a smoothing/derivative filter, (iii) normalization, (iv) spectral window selection, and (v) feature selection [37]. The data analysis implied the addition of multidimensional scaling (MDS) for data classification [20,21,22,23,24,25,26,38,57]. MDS calculations were developed from the similarities that analyzed spectra had in the dendrogram (results in Figures S1–S3 of the Supplementary Materials).

HCA is a statistical method that finds relatively homogeneous clusters of cases based on measured characteristics [22,23,24,25,26,38]. Such methodological analyses build tree-like groups by dividing or merging them successively. It starts with each case in a separate cluster and then combines the clusters sequentially, reducing their number at each step until only one cluster has been left [22,23,24]. A tree-like structure illustrates the arrangement of groups by hierarchical clustering [24,25,26,38]. 

Clustering algorithms and PCA have been widely implemented, yielding relevant and precise results for explorations of data in machine learning algorithms and extraction of safe information [66,67]. PCA involves the understanding of different features in a dataset and can be used in conjunction with cluster analysis [54,66,68,69,70,71,72,73]. It aims to map high-dimension data into low-dimension space [43,44,45,46,47,48,49,53,74,75,76]. It helped with the interpretation and understanding of the general behavior of the samples studied with no surplus overlapping, because the total number of spectra for each species was encapsulated in a PCA-center value. All PCA-center calculations and representations were performed by R Project for Statistical Computing [28,29,30,31,32,33,34,35,36].

Principal components–linear discriminant analysis correctly distinguished several degrees of drug-resistant strains, thus opening possibilities for the discovery of drug targets in *S. aureus* by SERS combined with PCA [7]. A classification based on latent structure discriminant analysis provided spectral variability directly [14]. The high sensitivity of SERS not only quantitatively distinguishes antibiotic-susceptible strains but also diagnoses the molecular targets of different antibiotics on bacteria [7]. 

An accurate identification of bacteria at resistance levels by FTIR can be challenging. Spectra of different bacterial species are known to be quite similar; therefore, FTIR is often used as an auxiliary tool in those cases. However, the identification of antimicrobial resistant bacteria shows a typical combination of lipid, Protein, and Carbohydrate spectra. The construction of machine learning algorithms can help to deal with characterizing biochemical profiles for the identification of MRSA.

### 4.4. Microorganism

*Staphylococcus aureus* strain (ATCC 25923) was cultured aerobically in a Brain Heart Infusion (BHI) liquid medium overnight at 37 °C and 150 rpm. The inoculum was centrifuged at 3000 rpm, and the pellet suspended in phosphate buffer saline (PBS) was centrifuged again. The inoculum was standardized at 10^7^ to 10^8^ colony-forming units per milliliter (CFU/mL) by optical density at 600 nm, and resistance was induced by amoxicillin, erythromycin, and gentamicin by cultivation in 6 mL of MH medium with an antibiotic concentration of ¼ MIC for 24 h at 37 °C, 150 rpm. Subsequently, the inoculum was centrifuged at 300 rpm and suspended in MH for standardization 10^8^ CFU/mL. The procedure was repeated for 3 cycles, and after resistance had been induced, the bacteria were plated in BHI agar.

### 4.5. Fourier Transformation Infrared Spectroscopy

Colonies from the plated samples at 37 °C for 24 h were collected for analysis by Attenuated Total Reflection (ATR) on an Agilent Cary 630 FTIR Spectrometer^®^ instrument. Colonies were evenly distributed over the crystal surface. A dry sample was scanned 250 times and recorded with a resolution of 4 cm^−1^, accumulating 16 scans per spectra. The result was the average of the measurements. The FTIR spectrum was measured in the 4000 cm^−1^ to 650 cm^−1^ wavelength range in all samples.

## 5. Conclusions

This study demonstrated that spectral analysis in the infrared regime enables the determination of whether a sample of bacteria is resistant to antibiotics and the antibiotic that has probably developed resistance in specific bacteria. This possibility goes beyond the detection of resistance, because it allows for evaluating the antibiotics that have contributed the most to this, giving the chance to understand the entire process of developing these resistances. Future studies with this technique will be of high relevance to the topic. 

## Figures and Tables

**Table 1:** Classification results for antibiotic identification with MC computation applied to FTIR absorption spectra data from hidden samples. Accuracy, sensitivity, and sensitivity matrix confusion calculations in the three windows intervals, Carbohydrates, Fatty Acids, and Proteins, in FTIR absorption spectra samples are presented.

**Hidden Sample**	**Window Group**	**Accuracy**	**Sensitivity**	**Specificity**	**Sample Susceptivity**
SK	Carbohydrates	0.938	0.95	0.987	ERY
	Fatty Acids	0860	0.8	0.920	
	Proteins	0.704	0.745	0.861	
SW	Carbohydrates	0.812	0.752	0.957	AMO
	Fatty Acids	0.714	0.767	0.936	
	Proteins	0.818	0.7	0.960	
SX	Carbohydrates	0.767	0.701	0.941	Control
	Fatty Acids	0.775	0.65	0.901	
	Proteins	0.832	0.75	0.915	
SY	Carbohydrates	0.915	0.87	0.962	MRSA
	Fatty Acids	0.933	0.9	0.996	
	Proteins	0.715	0.833	0.916	
SZ	Carbohydrates	0.941	0.91	0.972	GEN
	Fatty Acids	0.904	0.86	0.948	
	Proteins	0.818	0.733	0.960	

**Table 2:** CM identification results of antibiotic resistance of five hidden samples of *S. aureus*. The calculations were developed from FTIR absorption spectra acquired for the study.

**Hidden Sample Name**	**Real Resistance**	**Detected Resistance**	**Resistance to**	
1. SK	Resistance	Resistance	ERY	Correct
2. SW	Resistance	Resistance	AMO	Correct
3. SX	No Resistance	No Resistance	NONE	Correct
4. SY	Resistance	Resistance	MRSA	Correct
5. SZ	Resistance	Resistance	GEN	Correct

## Data Availability

Data available by request due to restrictions, e.g., privacy or ethical. The data presented in this study are available by request from the corresponding authors. The data are not publicly available due to the original data (nine hundred FTIR absorption spectra) being analyzed in another window of interest in the authors’ research group. Currently, the authors are developing research that involves the original data in their research group. The authors would like to mention that for this work no novel software was developed. In the Supplementary Materials, the authors supply a section with the steps implemented for their own code created to perform the data analysis in this article with the purpose of this information being useful to researchers and code developers. The authors sincerely want to encourage the researchers interested to contact them in case information is required about the process. To provide the support, the authors are able to be contacted by the email provided here.

## Supplementary Materials

The following supporting information can be downloaded at: https://www.mdpi.com/article/10.3390/antibiotics12101502/s1, Figures S1–S7: Dendrograms of entire sample FTIR spectra of *S. aureus* for four species studied, PCA-center, Confusion matrix results of identified and classified resistance to antibiotic induced sample from FTIR absorption spectra in the hidden samples SK into Carbohydrates interval window for the species: Control, AMO, ERY, GEN; Detailed Author Contributions.
