# Supplementary material for: Spectroscopic Identification of Bacteria Resistance to Antibiotics by Means of Absorption of Specific Biochemical Groups and Special Machine Learning Algorithm
Source: Antibiotics (Basel). 2023 Sep 30;12(10):1502. doi: 10.3390/antibiotics12101502 (PMC10604181; doi:10.3390/antibiotics12101502)
Supplement: Supplementary file 1 [file antibiotics-12-01502-s001.zip › antibiotics-2590586-supplementary.pdf]

Supplementary of the Paper (Article)

Spectroscopic Identification of Bacteria Resistance to Antibiotics by means of Absorption of Specific Biochemical Groups and Special Machine Learning Algorithm

Claudia P. Barrera-Patiño [1,*], Jennifer M. Soares [1], Kate C. Branco [1], Natalia M. Inada [1], Vanderlei Salvador Bagnato [1,2,*]

[1] São Carlos Institute of Physics, University of São Paulo, Avenida Trabalhador São-carlense nº 400, Parque Arnold Schimidt, São Paulo, São Carlos CEP 13566-590, Brazil

[2] Biomedical Engineering, Texas A&M University, 400 Bizzell St, College Station, TX 77843, United States of America.

* Correspondence: cpbarrerap@ifsc.usp.br, bagnatovs@tamu.edu

I. Spectra data analyses with machine learning unsupervised algorithms

1. Dendrogram calculation for Carbohydrates, Fatty Acids and Proteins windows into FTIR spectrum results for Control, Amoxicillin-induced (AMO), Gentamicin-induced (GEN), Erythromycin-induced (ERY).

Here we reported the use the Euclidean distance and single-linkage method to construct a dendrogram model used to developed the study in order to be implemented for the separation of antibiotic resistant and sensitive *S. aureus* strains [1], with the purpose to selected FTIR absorption spectra as inputs (features) for the classifiers Principal component analysis (PCA) in R [2–11], which allows that one multidimensional dataset will be simplified into several PCs to identify and distinguish various spectral groups with similar spectra [11,12].

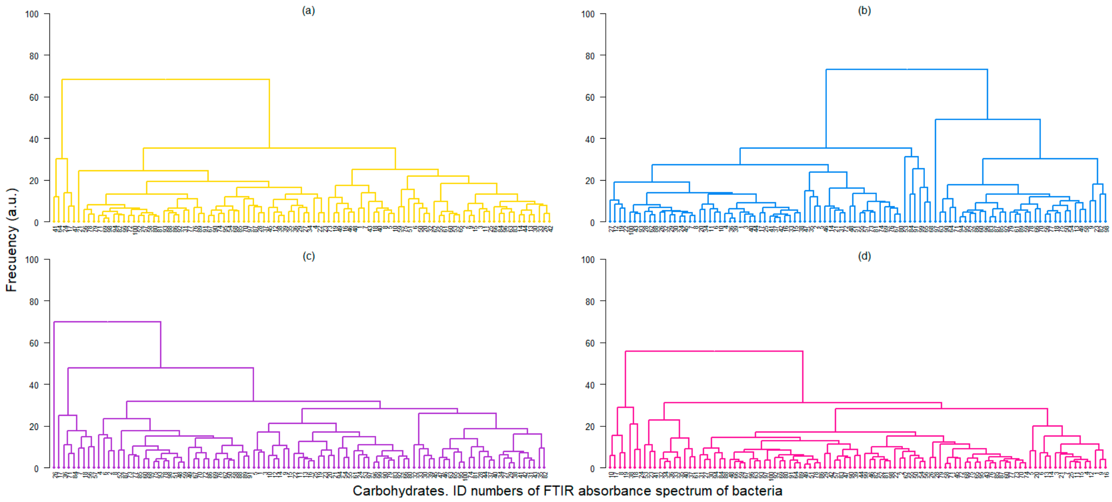

Figure S1. Dendrogram representation results for each one of one hundred FTIR spectrum into the Carbohydrates window for the four species groups a) Control, b) Amoxicillin-induced, c) Gentamicin-induced and d) Erythromycin-induced.

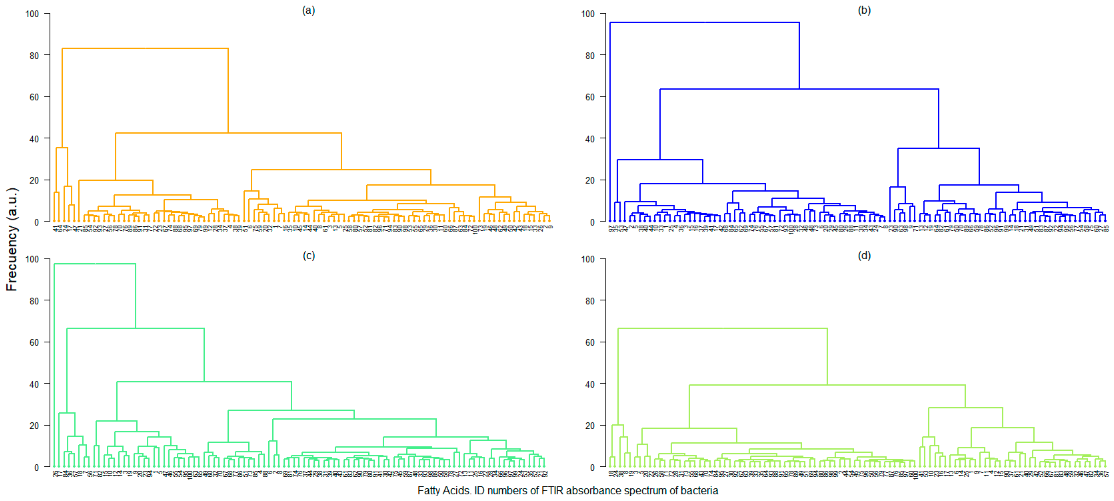

Figure S2. Dendrogram representation results for each one of one hundred FTIR spectrum into the Fatty Acids window for the four species groups a) Control, b) Amoxicillin-induced, c) Gentamicin-induced and d) Erythromycin-induced.

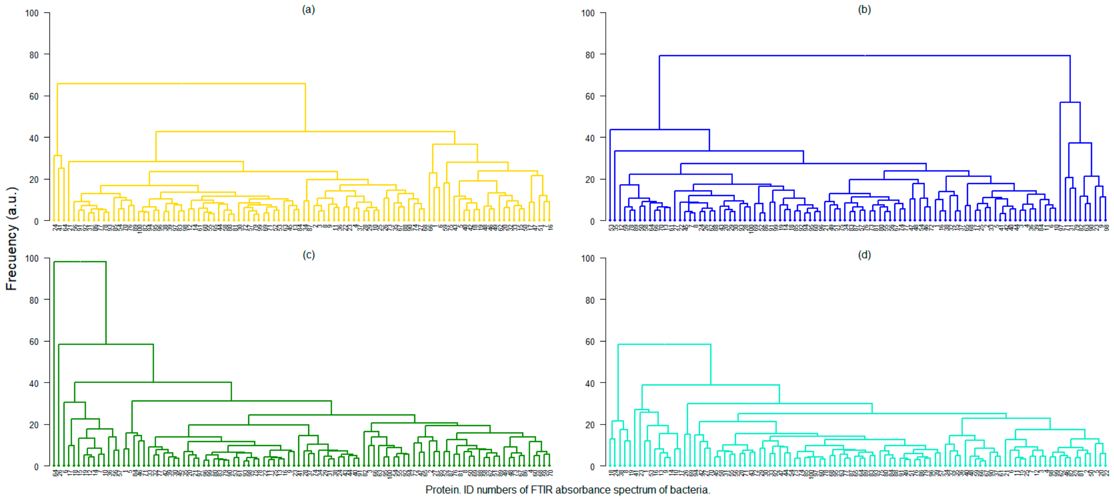

Figure S3. Dendrogram representation results for each one of one hundred FTIR spectrum into the Proteins window for the four species groups a) Control, b) Amoxicillin-induced, c) Gentamicin-induced and d) Erythromycin-induced.

2. PCA-center calculation results for Control, Amoxicillin-induced (AMO), Gentamicin-induced (GEN), Erythromycin-induced (ERY) into the three windows FTIR spectrum.

When all spectrum samples are process into the PCA-center we obtain the statistical representation and significance by scaling method of the FTIR spectrum. It lets to us to do the identification the representative results from PCA-center and spectrum are possible do the recognition of different behavior (figures 4-6). From the results we observed to the Carbohydrates spectrum window different intensity peaks shown in this region with a shape that differ one with another in absorbance intensity with the three windows studied for each four groups. In the case of Fatty Acids window, the intensity representation is obtained in a smaller values and number of picks and the distribution of them changes. To Proteins window also shown a particular shape and intensity values, that varies in relation with the previous windows.

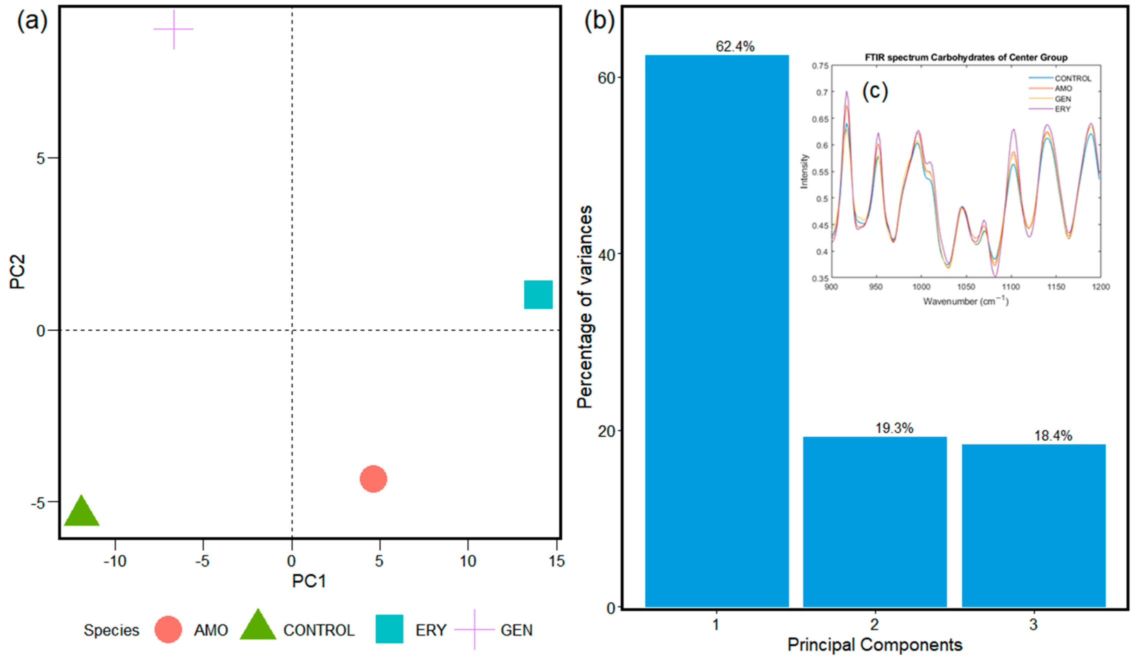

Figure S4. (a) PCA-center results for spectrum FTIR to Carbohydrate's window with (b) ~82% of statistical accuracy in first and second component in PCA-center (c). Spectrum center of one hundred FTIR data of *S. aureus* for each specie groups, Control, Amoxicillin-induced (AMO), Gentamicin-induced (GEN), Erythromycin-induced (ERY) into the window of Carbohydrates.

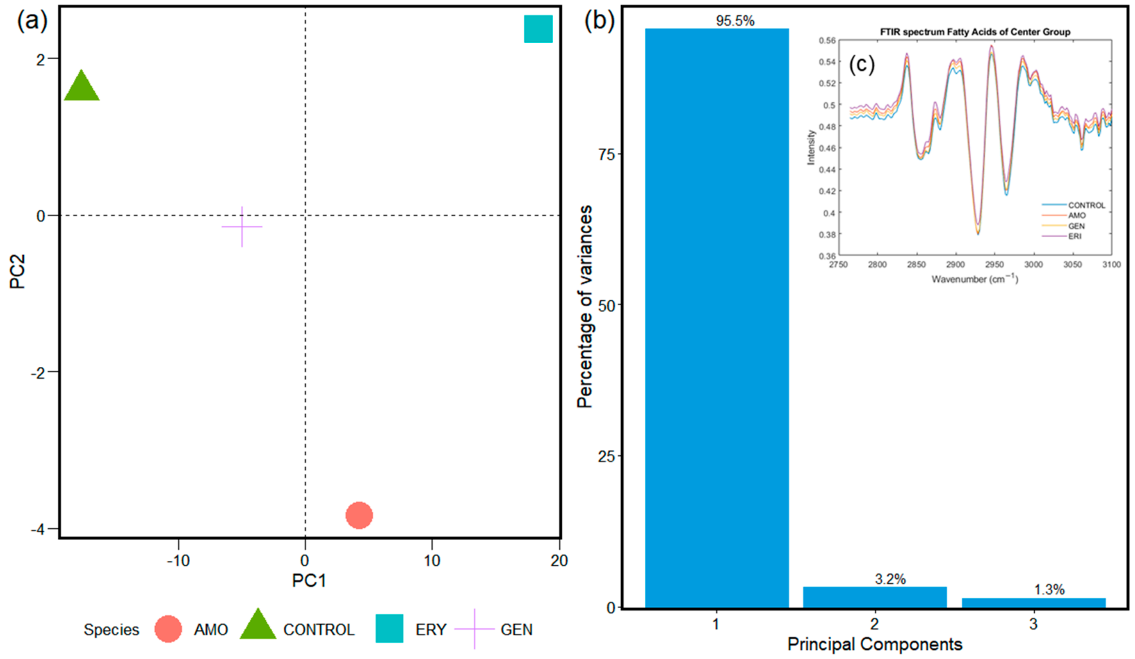

Figure S5. (a) PCA-center results for spectrum FTIR to Fatty Acids window with (b) 95,5% of statistical accuracy in first component in PCA-center. (c) Spectrum center of one hundred FTIR data of *S. aureus* for each specie groups, Control, Amoxicillin-induced (AMO), Gentamicin-induced (GEN), Erythromycin-induced (ERY) into the window of Fatty Acids.

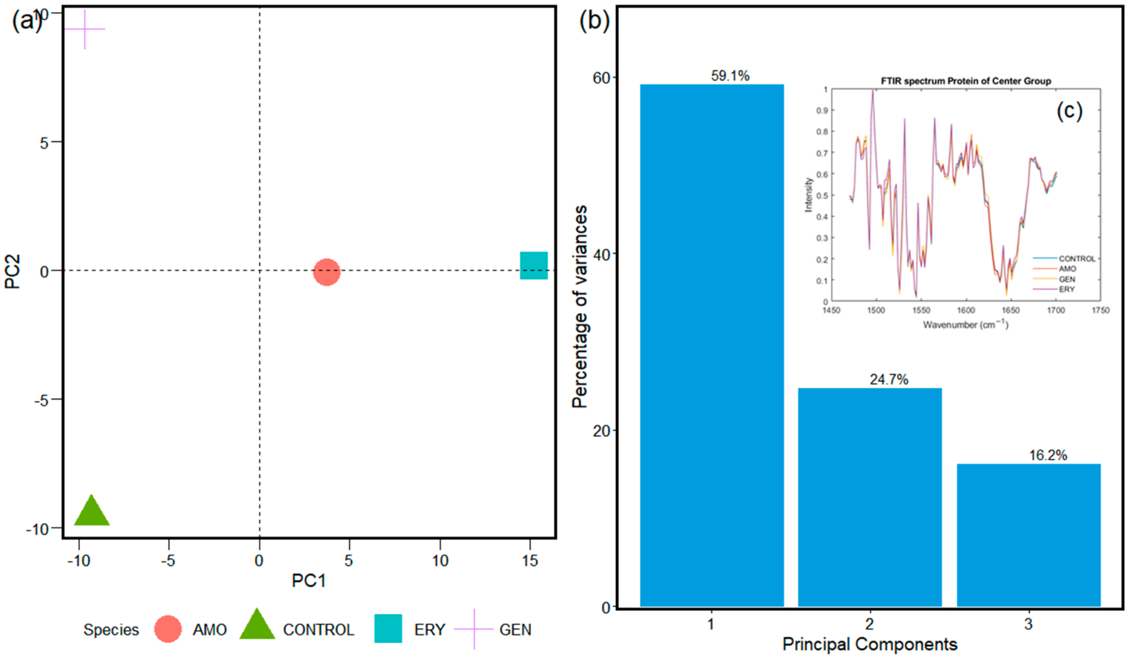

Figure S6. (a) PCA-center results for spectrum FTIR to Proteins window with (b) 83,8% of statistical accuracy in first and second component in PCA-center. (c) Spectrum center of one hundred FTIR data of *S. aureus* for each specie groups, Control, Amoxicillin-induced (AMO), Gentamicin-induced (GEN), Erythromycin-induced (ERY) into the window of Proteins.

II. Spectra data analyses with machine learning supervised algorithms

1. Confusion matrix results obtained from PCAs for the three windows.

The diagonal elements of this matrix show the number of correctly classified spectra, while the off-diagonal elements indicate the number of misclassified ones.

(a)

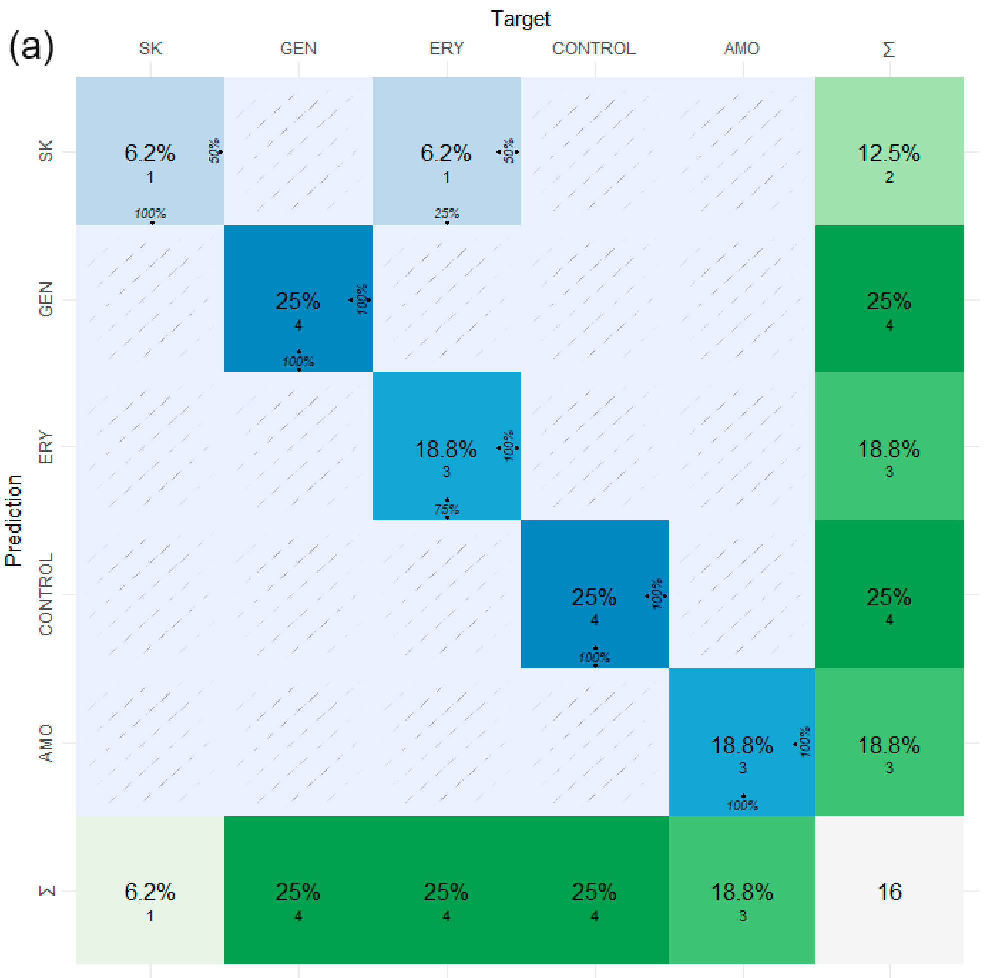

(b)

	target	prediction
	<fct>	<fct>
1	AMO	AMO
2	AMO	AMO
3	AMO	AMO
4	CONTROL	CONTROL
5	CONTROL	CONTROL
6	CONTROL	CONTROL
7	CONTROL	CONTROL
8	ERY	ERY
9	ERY	ERY
10	ERY	ERY
11	GEN	GEN
12	GEN	GEN
13	GEN	GEN
14	GEN	GEN
15	SK	SK
16	ERY	SK

(c)

`Overall Accuracy`	`Balanced Accuracy`	F1	Sensitivity	Specificity
<dbl>	<dbl>	<dbl>	<dbl>	<dbl>
0.938	0.968	0.905	0.95	0.987

Figure S7. Confusion matrix results to identified and classified resistance to antibiotic induced from FTIR absorption spectra in the hidden samples SK into Carbohydrates interval window for the species: Control, AMO, ERY, GEN. In this calculation where selected 80% of data to train the models join to 20% of the data for testing into MC. (a) CM plot results. (b) Prediction results table from CM with 0.938 of accuracy. (c) CM calculation table with statistical parameter: accuracy, sensitivity, sensibility, results obtained to hidden sample SK. This same process was done with all hidden samples studied here.

Author Contributions.

C. P. Barrera-Patiño. FTIR absorption spectra of *S. aureus* acquisition and record for the four species, ML algorithms tools implementation and develop of data classification, clustering, processing data and spectra data selection for the three windows groups in the four species groups, dimensionality reduction, data visualization, and antibiotic resistance sample evaluation and validation data analysis. Conceptualization. Participation in develop, determination and interpretation of data analyses results. Continual participation in results discussion reunion. Involvement into entire article texts. Writing the draft of the original text. Active participation and contribution in writing, edition and discussion about the text, methodologic contribution, and interpretation of results. Dynamic contribution in discussion section and conclusions about the research results.

Jennifer M. Soares. Antibiotic resistance *S. aureus* all sample preparation, FTIR absorption spectra acquisition and record for the four species and hidden samples. Collaboration with design of data analyses process to develop the spectra data analyses. Conceptualization. Continual

participation in discussion reunion about the results. She provided constant support to interpretation of results from data analyzes into the identification of antibiotic resistance (or not resistance) of FTIR spectra of *S. aureus* samples. Continual participation in results discussion reunion. Active participation and contribution in writing, edition and review the text. Helper in discussion and interpretation of results. Methodologic contribution, and interpretation of results. Dynamic contribution in discussion section and conclusions about the research results.

Kate C. Branco. Conceptualization. Continual participation in results discussion reunion. Active participation and contribution in review the text. Continuous talks about interpretation of results and suggestion to link the results with useful tools and applications developed into the study of biological systems.

Natalia M. Inada. Participation in discussion reunion. Resources. Validation. Visualization. Writing the original draft and review & editing article.

Vanderlei Salvador Bagnato. Idealization of research. Conceptualization. Methodologic steps developed helper, relevance classification help, ideas into the results evaluated in interpretation in all steps of job. suggestions of steps into the analyses data, it applied with success. Recommendation about inclusion of new methodologies and tools to be implemented into the analyses of FTIR spectra samples of *S. aureus*. Help in determination and interpretation of data analyses results in one way to improvement the visualization into the samples to determine with ML the similarities and differences between samples with and without antibiotic resistance. Continuous participation in discussion and evaluation of results. Involvement into entire article texts. Active participation and contribution in writing, edition and discussion about the text, methodologic contribution, and interpretation of results. Dynamic contribution in discussion section and conclusions about the research results. Continuous talks about interpretation of results and suggestion to link the results with useful tools and applications developed into the study of biological systems.

The authors confirm that all figures included in the Article main text and here in Supplementary Materials are results of the original work. The authors own all the material published here, and all the material is original results of the research submitted here.

References

1. Soares, J.M.; Guimarães, F.E.G.; Yakovlev, V. V.; Bagnato, V.S.; Blanco, K.C. Physicochemical Mechanisms of Bacterial Response in the Photodynamic Potentiation of Antibiotic Effects. *Sci. Rep.* **2022**, *12*, 21146, doi:10.1038/s41598-022-25546-y.
2. Nguyen, G.; Dlugolinsky, S.; Bobák, M.; Tran, V.; López García, Á.; Heredia, I.; Malík, P.; Hluchý, L. Machine Learning and Deep Learning Frameworks and Libraries for Large-Scale Data Mining: A Survey. *Artif. Intell. Rev.* **2019**, *52*, 77–124, doi:10.1007/s10462-018-09679-z.
3. Rizzo, M.L. *Statistical Computing with R*; CRC Press: Boca Raton, FL, USA, 2019
4. R Core Team. R: *A Language and Environment for Statistical Computing*, R Foundation for Statistical Computing, Vienna, Austria, 2021, https://www.R-project.org/.
5. R Core Team. R: *A Language and Environment for Statistical Computing*, R Foundation for Statistical Computing, Vienna, Austria, 2013, https://www.R-project.org/.
6. Mair, P.; Hofmann, E.; Gruber, K.; Hatzinger, R.; Zeileis, A.; Hornik, K. Motivation, Values, and Work Design as Drivers of Participation in the R Open Source Project for Statistical Computing. *Proc. Natl. Acad. Sci.* **2015**, *112*, 14788–14792, doi:10.1073/pnas.1506047112.
7. Fox, J. Aspects of the Social Organization and Trajectory of the r Project. *R J.* **2009**, *1*, 5.
8. Gareth, J.; Daniela, W.; Trevor, H.; Robert, T. *An Introduction to Statistical Learning: With Applications in R*; Spinger: Berlin/Heidelberg, Germany, 2013;
9. Chambers, J.M. *Software for Data Analysis: Programming with R*; Springer:

Berlin/Heidelberg, Germany, 2008; Vol. 2.

10. Ripley, B.D. The R Project in Statistical Computing. *MSOR Connect. Newsl. LTSN Maths Stats OR Netw*. **2001**, *1*, 23–25.
11. Wang, S.; Dong, H.; Shen, W.; Yang, Y.; Li, Z.; Liu, Y.; Wang, C.; Gu, B.; Zhang, L. Rapid SERS Identification of Methicillin-Susceptible and Methicillin-Resistant: Staphylococcus Aureus via Aptamer Recognition and Deep Learning. *RSC Adv.* **2021**, *11*, 34425–34431, doi:10.1039/d1ra05778b.
12. Wang, K.; Li, S.; Petersen, M.; Wang, S.; Lu, X. Detection and Characterization of Antibiotic-Resistant Bacteria Using Surface-Enhanced Raman Spectroscopy. *Nanomaterials* **2018**, *8*, doi:10.3390/nano8100762.
